# Antifungal and Antibacterial Activities of Apple Vinegar of Different Cultivars

**DOI:** 10.1155/2021/6087671

**Published:** 2021-08-07

**Authors:** Driss Ousaaid, Hassan Laaroussi, Meryem Bakour, Hayat Ennaji, Badiaa Lyoussi, Ilham El Arabi

**Affiliations:** ^1^Laboratory of Natural Substances Pharmacology Environment Modeling Health and Quality of Life, Faculty of Sciences Dhar El Mahraz, Sidi Mohamed Ben Abdellah University, Fez, Morocco; ^2^Division of Microbiology and Hygiene Products and Environment, Pasteur Institute, Casablanca, Morocco

## Abstract

This study was designed to assess the antimicrobial potencies of apple vinegar against pathogenic microbes. The acidity and total phenolic content were carried out by titration with NaOH 0.1 N and the Folin–Ciocalteu method, respectively, while the spread plate method, agar well diffusion, and MIC assays were used to determine the antimicrobial activities of different vinegar samples. Acidity and phenolic content were dependent on the variety, where the highest values were observed in S2 with 4.02 ± 0.04% and 1.98 ± 0.05 mg GAE/mL for acidity and total phenolic content, respectively. The spread plate method revealed that samples S1 and S2 obtained from the *Red delicious* variety and *Golden delicious* variety, respectively, inhibit the growth of all tested strains, while S3 obtained from different varieties and S4 obtained from the *Gala royal* variety inhibit only two microbes (*Escherichia coli* and *Vibrio cholerae*). Sample S1 presented moderate antimicrobial effect against all examined strains with a diameter of inhibition ranging from 11 ± 0.7 to 19 ± 0.5 mm and with MIC values ranging between 1/2 and 1/100. The findings of the current study confirm the usefulness of apple vinegar as a natural sanitizer that inhibits the growth of pathogenic microbes.

## 1. Introduction

The development of different methods used to produce food products is closely related to the reduction of infections caused by microorganisms. Moreover, food-borne epidemics continue to be a major public health problem. The profuse use of chemical antibacterial agents is harmful to human health and enhances the incidences of drug-resistant pathogens [1]. Natural products are healthy and safe products that offer antimicrobial effects and antioxidant properties simultaneously [2, 3].

Apple vinegar provides several pharmacological effects, for instance, antidiabetic effect [4–6], anti-Alzheimer effect [7], and antioxidant properties [2]. In addition, the administration of apple vinegar controls body weight gain and enhances glucose tolerance [8]. In experimental trials, the ability of apple vinegar as a natural product has been proved against human pathogens [9]. Thereafter, the sanitizing properties of vinegar have been reviewed in several studies. They reported that apple vinegar has an inhibitory effect against different bacterial strains such as *Staphylococcus aureus, Staphylococcus epidermidis*, *Staphylococcus pyogenes*, *Enterococcus faecalis, Streptococcus pneumoniae*, *Pseudomonas aeruginosa*, *Pseudomonas fluorescens*, *Escherichia coli, Salmonella typhi*, *Enterobacter aerogenes*, *Klebsiella pneumoniae*, *Proteus mirabilis*, *Proteus vulgaris*, and *Acinetobacter* [10, 11]. The remedial properties of apple vinegar are ascribed to its organic acids and its bioactive substances. It has been shown that organic acids pass into bacterial membranes which increases the synthesis of antimicrobial peptides, increases intern osmotic pressure, stimulates the consumption of energy, and sabotages macromolecular synthesis [12]. In addition to organic acids, apple vinegar contains other bioactive compounds that proved their antimicrobial potencies such as phenolic acids and flavonoids [3, 13–17].

In this vision, the present study was designed to determine the acidity and the phenolic content of different vinegar samples as well as their possible antimicrobial action against three bacterial pathogens and two fungal strains.

## 2. Materials and Methods

### 2.1. The Sampling of Apple Vinegar

The chosen samples of vinegar were produced by the artisanal process using three varieties of apples as presented in Table 1.

### 2.2. Bacterial and Fungal Strains

A total of five microbial strains, three bacterial strains, and two yeast isolates were used to examine the antimicrobial ability of different vinegar samples. The bacterial strains were represented by *Salmonella typhi* (CIP 5535)*, Escherichia coli* (CIP 54127), and *Vibrio cholerae* non-O1-non-O139 isolated from Tamoda Bay [18], while the tested yeasts were represented by *Candida albicans* (IPL CIM 861484) and *Candida tropicalis* (Pfizer CIM 4069).

### 2.3. Determination of Acidity and Total Polyphenolic Content (TPC)

The acidity of different studied samples was determined by titration with 0.1 N NaOH. Results were expressed as a percentage of acetic acid equivalent, while the quantification of the polyphenolic content was carried out using the Folin–Ciocalteu method. Results were expressed as mg of gallic acid equivalent per mL of vinegar (mg GAE/mL) [19].

### 2.4. Antimicrobial Assay

#### 2.4.1. Spread Plate Method

The test was performed by mixing 100 *μ*L of inocula diluted in physiological saline from broth grown overnight and 4 mL of each sample. The mixture was left for 5 min, and then 100 *μ*L of each mixture was inoculated onto an agar medium. The tests were replicated three times [20].

#### 2.4.2. Sensitivity Assay

*(1) Agar Well Diffusion Method*. The antimicrobial activity of different samples of vinegar was evaluated using the well diffusion method [10, 21]. The isolates were subjected to antimicrobial activity by the method mentioned above. *Salmonella typhi, Escherichia coli* O157:H7, and *Vibrio cholerae* were grown in the Tryptone Casein Soja (TCS) medium, and yeasts *Candida albicans* and *Candida tropicalis* were grown in Sabouraud Broth. 100 *μ*L of the active culture of different isolates consisting of 0.5 McFarland 1 *∗* 10^8^ CFU/mL was prepared in physiological saline. 40 *μ*L of the vinegar sample was placed in 5 mm diameter wells that have been cut in the agar of each Petri dish. Negative control wells were filled with sterile physiological water. Petri dishes were incubated at 37°C for 24 h for bacterial strains and at 30°C during 24–48 h for yeasts. Thereafter, the diameter of inhibition zones (DIZ) was measured.

#### 2.4.3. Minimum Inhibitory Concentration Assay (MIC)

The MIC was determined using a microdilution method according to the NCCLS method [21]. Firstly, dilution series of vinegar samples were prepared (S (initial solution), 1/2, 1/4, 1/6, 1/8, 1/10, 1/12, 1/100, and 1/150). Ten *μ*L of each prepared dilution was added to 180 *μ*L of TCS broth and 10 *μ*L of suspension of the active culture of different microorganisms tested (1 *∗* 10^8^ CFU/mL) in microplate wells. The plates were incubated at 37°C for 20 h for bacterial strains and were incubated at 30°C during the same time for yeasts. To reveal the growth of different studied microorganisms, we added to each well 20 *μ*L of the aqueous solution of 0.5% TTC (2,3,5-triphenyltetrazolium chloride), and then plates were incubated at 37°C for 30 min. The lowest dilution with no growth observed was defined as MIC (disappearance of red color after TTC addition) [22].

### 2.5. Statistical Analysis

ANOVA followed by the Tukey test was used for statistical analysis (*P* < 0.05) to show if there is any significant difference between samples.

## 3. Results

### 3.1. Sample Characterization

Table 2 represents the results of acidity and total phenolic content of different vinegar samples. It is shown that sample S1 had the highest acidity (4.02 ± 0.04%), while the lowest acidity value was registered in sample S4 (0.78 ± 0.07%). Concerning TPC, the highest value (1.98 ± 0.05 mg GAE/mL) was recorded in sample S1, while S4 recorded the lowest value (0.47 ± 0.06 mg GAE/mL).

## 4. The Antimicrobial Ability of Apple Vinegar

### 4.1. Results of the Spread Plate Method

The results of the spread plate revealed that the growth of different organisms tested was not observed in plates in the presence of samples S1 and S2 as presented in Table 3. The outcomes indicate that samples S1 and S2 were the most effective against all microbes because they inhibited the growth of all tested microorganisms.

### 4.2. Results of the Sensitivity Assay

Concerning antimicrobial activity, the vinegar samples were found to have a strong ability to inhibit the growth of microorganisms tested in the present work. Sample S1 produced the best antimicrobial effect against all strains with an inhibition diameter ranging between 12 and 19 mm, whilst sample S4 established the lowest antimicrobial activity. *Escherichia coli* O157:H7 was the most sensible bacterial strain. On the contrary, samples S3 and S4 presented the lowest activity against *Escherichia coli* O157:H7 and *Vibrio cholerae* with the lowest inhibition diameter ranging from 8 to 11 mm, respectively (Table 3). Statistically, there is a significant difference between the effect of vinegar samples studied on bacterial and yeast isolates.

Concerning examined yeasts in the present work, sample S1 was the most active on *Candida albicans* and *Candida tropicalis* with a diameter of inhibition ranging between 11 and 12 mm, whilst samples S2, S3, and S4 caused no inhibition of *Candida albicans* and *Candida tropicalis* as shown in Table 4.

In the present work, the MIC values are shown in Table 5. It was clearly shown that sample S1 exhibited remarkable antimicrobial activity against all microorganisms, with MIC ranging from S to 1/100, followed by sample S2. Amongst all microorganisms tested, *E. coli* was the most sensitive for all studied vinegar samples, while *Candida albicans* and *Candida tropicalis* were more resistant for S2, S3, and S4.

## 5. Discussion

Historically, humans have always sought to cure various diseases using different plants and products from the surrounding nature. The emergence of antimicrobial resistance has been paralleled with the misuse of antimicrobial drugs which induces serious problems for human health [23].

Natural products of antimicrobial potency are the best biorational and effective agents as useful bio-antimicrobial drugs [24]. The failure of antibiotics used to eradicate different pathogenic microbes prompted us to study the ability of vinegar to inhibit the growth of pathogenic microbes.

The outcomes revealed that the evaluated vinegar, especially sample S1, has a potent antimicrobial effect against both bacterial strains and fungal isolates. The analysis of results indicates that sample S1 had the highest acidity and phenolic content as compared with other samples (*P* < 0.05). The values of acidity recorded in different samples are lower than those determined by Moroccan legislation [25]. Furthermore, the obtained results are in line with previous reports [26, 27]. Acidity is a key factor to determine the quality of apple vinegar, and the minimum of vinegar acidity is set at 5 grams of acetic acid equivalent per 100 mL [25]. Organic acids' content determines the flavoring profile of vinegar and depends on the raw material [26]. In addition, vinegar's organic acids play a crucial role in different properties of vinegar such as antimicrobial activities, antidiabetic effects, and anticancer activities [6, 10, 11, 28].

Concerning the phenolic content, the highest value was found in S1. It is considered as an important criterion for quality evaluation [3]. The phenolic content values varied in the range of 1.98 ± 0.05 (S1) to 0.47 ± 0.06 mg GAE/100 mL (S4). These results are in agreement with those reported by Du et al. and Kim et al. [29, 30]. The polyphenolic content of apple vinegar is highly related to the variety of apples, maturity, and geographic location [31]. Secondary metabolites constitute a defense weapon of plants against different pathogenic agents such as fungi, viruses, and bacteria [32]. The efficacy of natural products could be related to their content of bioactive compounds which provide their broad spectrum of antimicrobial activities [33, 34].

Many pathogenic microbes can survive despite the use of antimicrobial chemicals. In the present study, we examined the ability of four vinegar samples to eradicate different pathogenic microbes (bacteria and yeasts). S1 exerted a good activity against all tested microbes with diameter zones ranging from 11 to 12 mm for yeasts and 16 to 19 mm for bacteria, while other samples showed weak antimicrobial activity. The antimicrobial activity could be due to the presence of organic acids, especially acetic acid. Sample S1 registered the highest value of acidity and showed remarkable antimicrobial potency. Our findings are partially coherent with the published studies reporting the efficacy of apple vinegar against *Salmonella* and *Escherichia coli* [35]*, Streptococcus pyogenes* [36]*, Staphylococcus aureus, Staphylococcus epidermidis, Streptococcus pyogenes, Enterococcus faecalis, Streptococcus pneumoniae, Pseudomonas aeruginosa, Pseudomonas fluorescens, Enterobacter aerogenes, Klebsiella pneumoniae, Escherichia coli, Salmonella typhi, Proteus mirabilis, Proteus vulgaris*, and *Acinetobacter* [10], *Aspergillus niger, Aspergillus flavus*, and *Candida albicans* [37].

In the literature, several studies showed the efficiency of organic acids against numerous microorganisms [38, 39]. The obtained results could be attributed to the presence of bioactive compounds such as organic acids and phenolic compounds [33, 39–42].

The mechanism of action was recently authored by Zhang et al. in which organic acids display residual effect to prevent the growth of pathogenic microbes [12]. The ability of organic acids to liberate protons H^+^ into cells decreases intracellular pH, inducing the destruction of bacteria membrane cells [12]. On the contrary, the high levels of hydrogen ions lead to the protonation of cell macromolecules destabilizing the microbes and inducing their death [12]. The expulsion of protons is carried out via active transport which consumes the energy necessary for the normal growth of microbes [13]. In the same context, phenolic compounds present in vinegar could participate in the inhibition processes of microbe's growth. Polyphenols could disturb the integrity of membrane cells which modify their permeability [42] and decrease extracellular pH [43]. Bioactive compounds interact with different intramolecular ingredients of cells [44], forming complexes and affecting the processes of energy production and protein synthesis [45].

Furthermore, the interaction between bioactive compounds and organism's cell surfaces is liable for the antimicrobial effect of vinegar [42]. It was found that apple vinegar contains several phenolic compounds such as vanillic acid, chlorogenic acid, caffeic acid, gallic acid, catechin, epicatechin gallate, and phlorizin [29]. It has been documented that chlorogenic acid effectively alleviates lung infection in *Klebsiella pneumonia*-infected mice and provides a remarkable antibacterial activity [46]. Similarly, Carvalho et al. showed that tannin and gallic acid are suggested to be antimicrobial agents through the inhibition of catalase and binding membrane ergosterol [47]. The cocktail composition of vinegar provides it the ability to inhibit the growth of different organisms through the synergy between its active ingredients which confirm their antimicrobial potencies.

## 6. Conclusion

In the present work, the evaluated apple vinegar samples, especially S1, demonstrated an adequate antimicrobial potency against different studied strains. Functional properties of apple vinegar could be related to the presence of organic acids and phenolic compounds. Vinegar, as an organic product, could be used as a natural sanitizer and also as a bioactive ingredient in the food industry.

## Figures and Tables

**Table 1:** The sampling of apple vinegar.

Samples	Source	Varieties of apple	Stations
S1	Herbalist Iklil Al-Jabal	*Red delicious*	Midelt
S2	Herbalist Iklil Al-Jabal	*Golden delicious*	Midelt
S3	Cooperative Al Jazeera	Different varieties	Midelt
S4	Cooperative Domaine Chène Vert	*Gala royal*	Sefrou

**Table 2:** Acidity and polyphenolic content in apple vinegar samples.

Sample	Acidity (%)	TPC (mg GAE/mL)
S1	4.02 ± 0.04	1.98 ± 0.05
S2	2.6 ± 0.5	1.3 ± 0.0.03
S3	1.4 ± 0.09	0.88 ± 0.01
S4	0.78 ± 0.07	0.47 ± 0.06

**Table 3:** Results of microorganisms and vinegar samples in the spread plate method.

Organism	In the presence of vinegar				In the presence of physiological water
	S1	S2	S3	S4	
*Salmonella typhi*	No growth	No growth	Growth	Growth	Growth
*Escherichia coli* O157:H7	No growth	No growth	No growth	No growth	Growth
*Vibrio cholerae*	No growth	No growth	No growth	No growth	Growth
*Candida albicans*	No growth	No growth	Growth	Growth	Growth
*Candida tropicalis*	No growth	No growth	Growth	Growth	Growth

**Table 4:** Diameters of inhibition zones (DI) of vinegar samples.

Organism		Diameter of the inhibition zone (mm)			
		S1	S2	S3	S4
Bacteria	*Salmonella typhi*	15 ± 0.3	14 ± 0.9	No effect	No effect
	*Escherichia coli* O157:H7	19 ± 0.5	13 ± 0.6	8 ± 0.3	8 ± 0.7
	*Vibrio cholerae*	16 ± 0.8	14 ± 0.4	11 ± 0.5	9 ± 0.2
Yeast	*Candida albicans*	12 ± 0.5	No effect	No effect	No effect
	*Candida tropicalis*	11 ± 0.7	No effect	No effect	No effect

**Table 5:** Minimum inhibitory concentration (MIC) of vinegar samples.

Sample	*S. typhi*	*E. coli*	*V. cholerae*	*C. albicans*	*C. tropicalis*
S1	1/6	1/100	1/2	1/2	1/2
S2	S	S	S	No effect	No effect
S3	No effect	S	No effect	No effect	No effect
S4	No effect	S	No effect	No effect	No effect

## Data Availability

The data used to support the findings of this study are included within the article.
